# Co-sonicated coacervation for high-efficiency green nanoencapsulation of phytosterols by colloidal non-biotoxic solid lipid nanoparticles

**DOI:** 10.1038/s41598-024-54178-7

**Published:** 2024-02-26

**Authors:** Zolfaghar Mahdlou, Rahebeh Amiri Dehkharghani, Ali Niazi, Atefeh Tamaddon, Maryam Tajabadi Ebrahimi

**Affiliations:** 1 Department of Chemistry, Central Tehran Branch, Islamic Azad University, P.O. Box 1465613111, Tehran, Iran; 2 Department of Biology, Faculty of Sciences, Central Tehran Branch, Islamic Azad University, Tehran, Iran

**Keywords:** Plant sciences, Nanoscience and technology

## Abstract

Plant sterols are used as a supplement or an additive to reduce LDL cholesterol. The poor dispersibility and instability of phytosterols are the main limitations of their application. So, we tried to overcome these problems through nanoencapsulation of them with colloidal natural RSs (SLNs) using an effective approach to achieve higher efficiency and less intrinsic coagulation. Phytosterols extracted from flax seeds oil with caffeine by a new method were encapsulated with a stable colloid of sheep fat and ostrich oil (1:2), soy lecithin, and glucose through co-sonicated coacervation. Characterization of the obtained SLNs was conducted using FTIR, UV–Vis, SEM, DLS, and GC analysis. The three–factor three–level Behnken design (BBD) was used to prioritize the factors affecting the coacervation process to optimize particle size and loading capacity of SLNs. Operational conditions were examined, revealing that the size of SLNs was below 100 nm, with a phytosterols content (EE %) of 85.46% with high positive zeta potential. The nanocapsules' anti-microbial activity and drug-release behavior were then evaluated using the CFU count method and Beer-Lambert's law, respectively. The controlled release of nanocapsules (below 20%) at ambient temperature has been tested. The stability of nano-encapsulated phytosterols was investigated for six months. All results show that this green optimal coacervation is a better way than conventional methods to produce stable SLNs for the nanoencapsulation of phytosterols.

## Introduction

Encapsulation is an expanding technology with potential applications in pharmaceutical and other industries. The methods can be employed to protect sensitive compounds (such as enzymes, polyphenols, antioxidants, and nutraceuticals) from environmentally destructive effects and also for the controlled delivery at targeted sites^1,2^ in the range of micro or nano diameter. In this among, nanocapsules are vesicular systems where a core compound can be confined to a cavity covered by a special membrane. SLN as one of these coating components are potential colloidal systems which capable of carrying especially lipophilic drugs, such as plant sterols. However, this carrier has low drug loading efficiency and uncontrolled particle growth through coagulation or agglomeration^3^. On the other hand, plant sterols have many bioactive effects with human health advantages^4,5^ such as decreasing serum cholesterol concentrations^6^ preventing heart disease^7^, and anti-cancer effects^8^. Campesterol, β-sitosterol, stigmasterol, and ergosterol^9^ are members of the phytosterols family and their chemical structure is similar to cholesterol. Also, tocopherols and phytosterols are prominent nutrient components making up the most significant amount of the non-saponifiable fraction of vegetable oils^10^. However, their main limitation is their poor solubility in an aqueous medium (2–3% at 25 °C, w/w)^11^ and instability at high temperatures under airflow^12,13^. So, phytosterols are poorly absorbed by the human body, and a high dose is needed to absorb sufficient quantity^14^ they are also easily oxidized^15^, especially in food production and storage processes. Several techniques have been investigated to improve phytosterols’ dispersibility, such as high-pressure homogenization^16^. The microencapsulation of phytosterols was also studied using spray chilling with a lipid mixture^17^ or spray drying^18^. But none fully covered the rational design of a particulate system including phytosterols with sufficient consumer acceptability and bioavailability (nanoscale particle size or organic solvent free-product). Recently, coacervation was introduced as a new approach for the microencapsulation of lipophilic compounds such as essential oils^19^. The mechanism of the process consists of the separation of the hydrocolloid from the primary solution with a coacervating compound such as a desolvating agent (nonsolvent, pH change, temperature or electrolyte), or a crosslinking agent, such as glutaraldehyde^20^. In this work, we have directed our attention towards a non-liposomal coating that exhibits a natural structure and we have also given consideration to the dispersing of selected SLNs as a carrier for phytosterols.solid. So, the co-sonicated coacervation technique was employed to enhance both the distribution capability and stability of the nanoparticles. At first, an innovative green process under low temperatures with caffeine was used to extract the phytosterols instead of chemical saponification at reflux temperature to minimize damage to them. After that, the extracted phytosterols were covered with natural fats such as ostrich oil with many useful edible ingredients (Omega 9, 6, and 3), sheep fat, and lecithin (amphiphilic surfactant) instead of synthetic polymeric stabilizers^21^. We used for the first time, the amplified ultrasound coacervation to make a stable colloidal system for reducing the average particle size (nanometer scale) and increasing the loading capacity with minimal coagulation. Also, the response surface methodology (RSM) can be applied for optimization and modeling in many analytical processes^22,23^. Box–Behnken design (BBD), offers a balanced and efficient approach for modeling and optimizing complex systems, with reduced experimental runs and improved estimation accuracy compared to other RSM techniques^24^. Therefore it can be used for experimental design that can be used for nanoencapsulation^25^. So, in the present study, the impacts of independent factors on the nanoencapsulation process were evaluated and optimized by theoretical calculations. In addition to the study of antibacterial properties, the product stability was assessed within six months and the dependence of drug release behavior on temperature was investigated.

## Discussion and results

Caffeine was used to extract phytosterols based on the alkali nature of this substance which can form ionic salt with free fatty acids in flaxseed oil. On one side, caffeine has poor water solubility at room temperature, so we can recrystallize it by lowering the temperature^26,27^. On the other side, we used binary solvents for creating a balance between the hydrophilic and hydrophobic properties (water: ethanol, 50:50) to extract the phytosterols because caffeine is hydrophilic and doesn’t penetrate the organic phase (log *P* = − 0.07*)*. Consequently, the new crystals can be deposited and phytosterols remain in the organic phase. The *pKa*-value difference between ionizable molecules (caffeine and fatty acids) is less than 2 units which is required to achieve an acceptable stability level of the resulting salt^28,29^.

**Figure 1:**
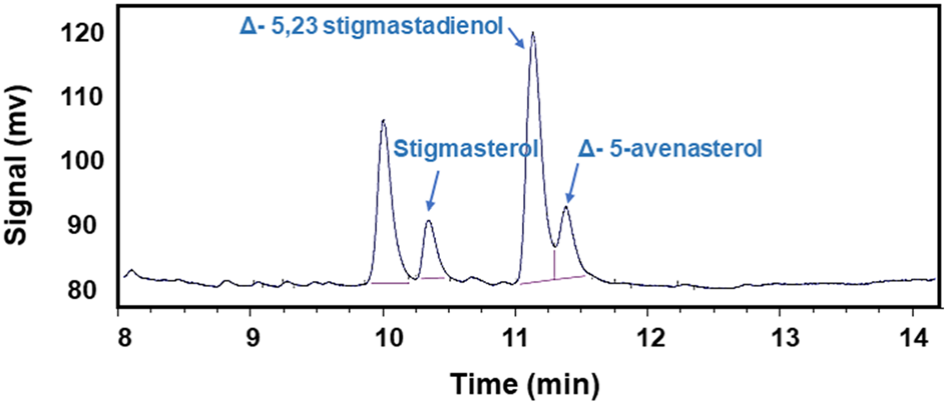
GC Chromatogram of extracted phytosterols.

**Scheme 1:**
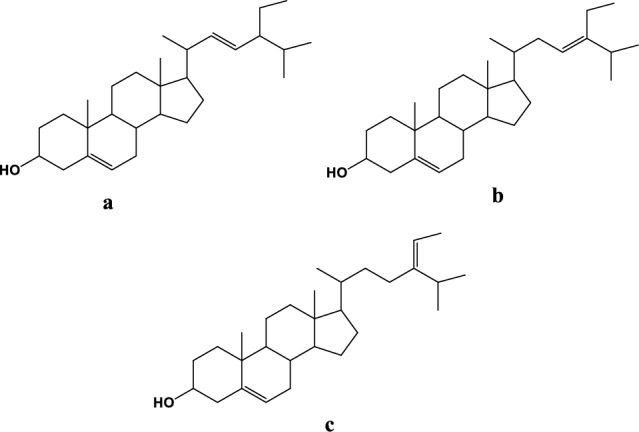
Structural formulas of (**a**) Stigmasterol, (**b**) ∆^5,23^-Stigmastadienol and (**c**) ∆^5^-Avenasterol.

Phytosterols’ composition and content were analyzed using GC analysis^30^. The GC peaks of sterols are shown as their retention times by the internal or reference standards. Validation methods for the determination of sterols such as quality control checking assessments using GC-FID were reported^31^. The chromatogram shows 3 identifiable phytosterols in (Fig. 1). The major component is ∆^5,23^-stigmastadienol (47%). The other different components may vary depending on the extraction method and oilseed type. Therefore, their values and types of phytosterols can be variable. The chemical structure of the main phytosterols in flaxseed oil is shown in (Scheme 1).

The used levels and ranges are selected at the base of the preliminary experiments. The experimental ranges related to independent variables were: sheep fat to ostrich oil proportion (X_1_: 1.0–2.0), percentage of glucose (X_2_: 40–60), amount of phytosterols (X_3_: 0.06–1.0), percentage of lecithin (X_4_: 6.0–10.0) and nanoencapsulation time (X5: 2.0–5.0). BBD was applied to optimize and assess the significant variables. In total, 17 tests containing three replicates at the center point were done randomly according to the Box–Behnken matrix, (Table 1).

**Table 1:** Factors and their levels in Box-Behnken design matrix and observed response.

Run No	The actual level of factors					
	X_1_	X_2_	X_3_	X_4_	X_5_	Y (Absorbance)
1	1.0	40	0.06	10	2.0	0.0604
2	2.0	60	1.00	6	2.0	0.3009
3	2.0	60	0.06	10	2.0	0.1072
4	2.0	60	1.00	10	5.0	0.0532
5	2.0	40	1.00	10	2.0	0.1054
6	1.0	60	1.00	6	5.0	0.2585
7	2.0	40	0.06	10	5.0	0.0886
8	1.0	40	1.00	6	2.0	0.2081
9	1.0	40	1.00	10	5.0	0.1489
10	1.0	50	0.53	8	3.5	0.1891
11	1.0	60	0.06	6	2.0	0.5518
12	2.0	60	0.06	6	5.0	0.0519
13	2.0	40	0.06	6	2.0	0.0726
14	1.0	60	1.00	10	2.0	0.0262
15	1.0	60	0.06	10	5.0	0.0818
16	2.0	40	1.00	6	5.0	0.2551
17	1.0	40	0.06	6	5.0	0.0915

X_1_: sheep fat: ostrich oil (1:2 X_1_ = 1, 2:1 X_1_ = 2); X_2_: percentage of glucose;

X_3_: the amount of phytosterols (g); X_4_: percentage of lecithin and X_5_: nanoencapsulation time (hr.).

The relationship between responses and input variables, the data were fitted to a second-order polynomial mathematical equation. The analysis of variance (ANOVA) determined the significance of the model and its terms. As presented in (Table 2), the significance of the terms was assessed using their *p*-values.

**Table 2:** Analysis of variance (ANOVA) evaluation of linear, quadratic, and interaction terms for each response variable.

Variables	SS^a^	MS^b^	F-values	*p*-value
Model	0.9812	0.0141	23.21	0.000
X_1_	0.0127	0.0813	48.26	0.000
X_2_	0.0341	0.0714	57.25	0.001
X_3_	0.0205	0.0352	74.06	0.000
X_4_	0.0419	0.0568	0.89	0.000
X_5_	0.0213	0.0147	1.89	0.213
X_1_^2^	0.0187	0.0638	45.23	0.000
X_2_^2^	0.0021	0.0213	36.79	0.000
X_3_^2^	0.0471	0.0142	19.75	0.000
X_4_^2^	0.0255	0.0874	57.14	0.000
X_5_^2^	0.0451	0.0245	36.45	0.000
X_1_X_2_	0.0187	0.0458	36.45	0.000
X_1_X_3_	0.0142	0.0145	65.14	0.000
X_1_X_4_	0.0028	0.0398	18.49	0.003
X_1_X_5_	0.0391	0.0098	24.32	0.751
X_2_X_3_	0.0088	0.0008	68.12	0.002
X_2_X_4_	0.0045	0.0098	36.45	0.000
X_2_X_5_	0.0291	0.0082	45.87	0.387
X_3_X_4_	0.0008	0.0003	36.12	0.985
X_3_X_5_	0.0015	0.0251	4.23	0.368
X_4_X_5_	0.0171	0.0020	6.02	0.000
Residual	0.0036	0.0009		
Lack-of-fit	0.0048	0.0068	2.12	0.402
Pure error	0.0093	0.0003		

R^2^ = 99.23; Predicted R^2^ = 98.36; Adjusted R^2^ = 99.08; ^a^SS: sum of squares; ^b^MS: mean square.

Based on the results obtained from Tables 1 and 2, it can be observed that the method under consideration was significantly influenced by three factors including the quantity of phytosterols, the glucose percentage, and the proportion of sheep fat to ostrich oil, respectively. The Pareto chart to confirm the influence of these various factors on the impact factor has been shown in the supplementary Fig. 1.

The following statistically significant model only with significant terms was written (Eq. (1)):

1 $$ \begin{aligned} {\text{Y}} = & 0.{8941} + {37}.{\text{25X}}_{{1}} + 0.{\text{45X}}_{{2}} + 0.{\text{15X}}_{{3}} + {1}.0{\text{5X}}_{{4}} + {1}.0{\text{6X}}_{1}^{2} + 0.0{\text{24X}}_{3}^{2} + 0.{\text{78X}}_{4}^{2} + 0.0{\text{19X}}_{{1}} \times {\text{X}}_{{2}} \\ & + 0.{\text{74X}}_{{1}} \times {\text{X}}_{{3}} + 0.0{\text{9X}}_{{1}} \times {\text{X}}_{{4}} + 0.{\text{21X}}_{{2}} \times {\text{X}}_{{4}} + 0.{\text{47X}}_{{3}} \times {\text{X}}_{{4}} \\ \end{aligned} $$

The critical points in the surface response can be detected by solving the derivation of achieved Eq. 2 for the condition of δ(Y)/δ(X1) = 0, δ(Y)/δ(X2) = 0, δ(Y)/δ(X3) = 0, δ(Y)/δ(X4) = 0 and δ(Y)/δ(X5) = 0 this model. The obtained values for the critical point for nanoencapsulation of phytosterols are X1 = 0.9, X2 = 55, X3 = 0.06, X4 = 6, and X5 = 2.

The ANOVA of the regression model indicates that the model was highly significant at *P* < 0.05. The adjusted determination coefficient (adjusted-R^2^) and coefficients of determination (R^2^) were used to evaluate the quality of the fitted model. A regression model (R^2^ > 0.95) exhibits a very high correlation. The very high R^2^ value (0.9923) represented an excellent correlation between predicted and experimental values and this model described 99.23% variability of the response. Furthermore, the adjusted-R^2^ (0.9836) was also very high and confirmed the model's high significance. The lack of fit test can measure the model's failure to indicate experimental data in the experimental domain at the points not considered in the regression analysis. Lack of fit was non-significant indicating the significance of the quadratic model for the response.

**Figure 2:**
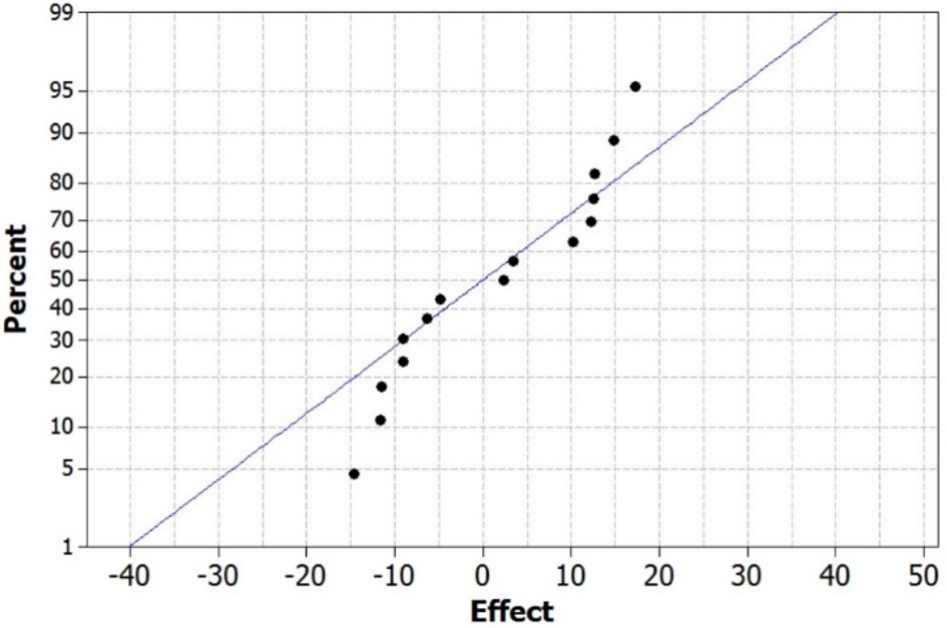
The normal probability plot of residuals.

The normal distribution of data is necessary for the statistical analysis of the experimental data, which can be checked by plotting a normal probability plot of the residuals (Fig. 2). Based on the Anderson–Darling test, a residual distribution was assessed for normality. Figure 2 indicates that the points will follow a straight line and residuals follow a normal distribution. So, none of the RSDs isn’t higher than 1 number, and the *p*-value for the test (0.316) approved this result.

**Figure 3:**
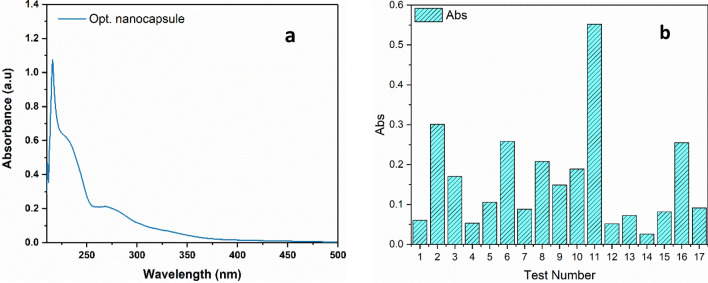
UV–Vis spectrum of the nano-encapsulated phytosterols (**a**) and Comparative absorbance of 17 synthesized samples (**b**).

UV–Vis absorption spectra of the final nano-encapsulated phytosterols (1mg/mL) in hexane were taken in the presence of the blank (without phytosterols). The maximum absorption peak of nano-encapsulated phytosterols is at 208 nm (π → π* transition). Also, some absorption peaks have appeared at higher wavelengths which can be related to transition n → π* of the –OH group (Fig. 3a). Furthermore, there is a chart for comparing different absorptions of seventeen tests which were designed by Box Behnken optimization. Run **11** in (Fig. 3b) with the highest absorption has shown the best possible formulation in terms of absorbance. The values selected for each of the factors influencing the process have been listed in (Table 1). The values in run **11** for sheep fat: Ostrich oil is 1:2, glucose is 60%, phytosterols are 0.06 g, lecithin is 6% and the necessary time to form a homogeneous mixture is 2 h.

On the other hand, calibration curve of the phytosterols concentration has been studied using the known concentration of them in 10 mL n-hexane at 208 nm (It is available in the supplementary Fig. 2). To determine the phytosterols content in the final nanocapsules, the concentration from the UV–Vis measurement was reported relative to the initial phytosterols concentration by division. The phytosterols content or entrapment efficiency (EE%) of the product was calculated to be 85.46% in optimum condition according to the following formula (Eq. (2)).

2 $$ {\text{EE}}\% = \left( {\text{Weight of drug in nanoparticles}} \right)/\left( {\text{Weight of drug fed initially}} \right) \times {1}00 $$

Dynamic light scattering (DLS) was used to determine the particle size distribution of the obtained nanoparticles. The final product (with an appropriate amount) was dispersed in distilled water (total concentration: 1%). Particle size affects drug release, cellular uptake, and physical stability. In this work, size distribution is affected by the co-sonicated coacervation method and the different variables designed by the BBD. In the process, we encountered a kind of complex coacervation which is a phase separation due to the interaction of some oppositely charged colloids (salt of fatty acids, phytosterol, lecithin, and glucose). The main driving force for the complex coacervation can be a reduction in free electrostatic energy between oppositely charged ions or a change in some parameters such as pH (It should be exactly below the isoelectric point), the concentration of core compound, the ratio of other materials which drives the coacervation process and results in the formation of coacervates^20^. On the other hand, complex coacervates exhibit remarkably low interfacial tensions, which play a crucial role in their stability and distinctive characteristics. These reduced interfacial tensions have the potential to enhance the encapsulation of active constituents^32^. In general, the effects of agitation and ultrasonic waves are evidenced by the inhibition of the formation of significant aggregates, the facilitation of dispersion, and the enhancement of droplet fragmentation. By manipulating these variables, it is possible to control the coacervation process and subsequently impact the distribution of particle sizes. So, we qualified the coacervation with ultrasonic waves to prevent the intrinsic coagulation of SLNs during the formation and the BBD was applied to optimize all factors (quantity of phytosterols, the glucose percentage, and the proportion of sheep fat to ostrich oil) that are effective in this process.

**Figure 4:**
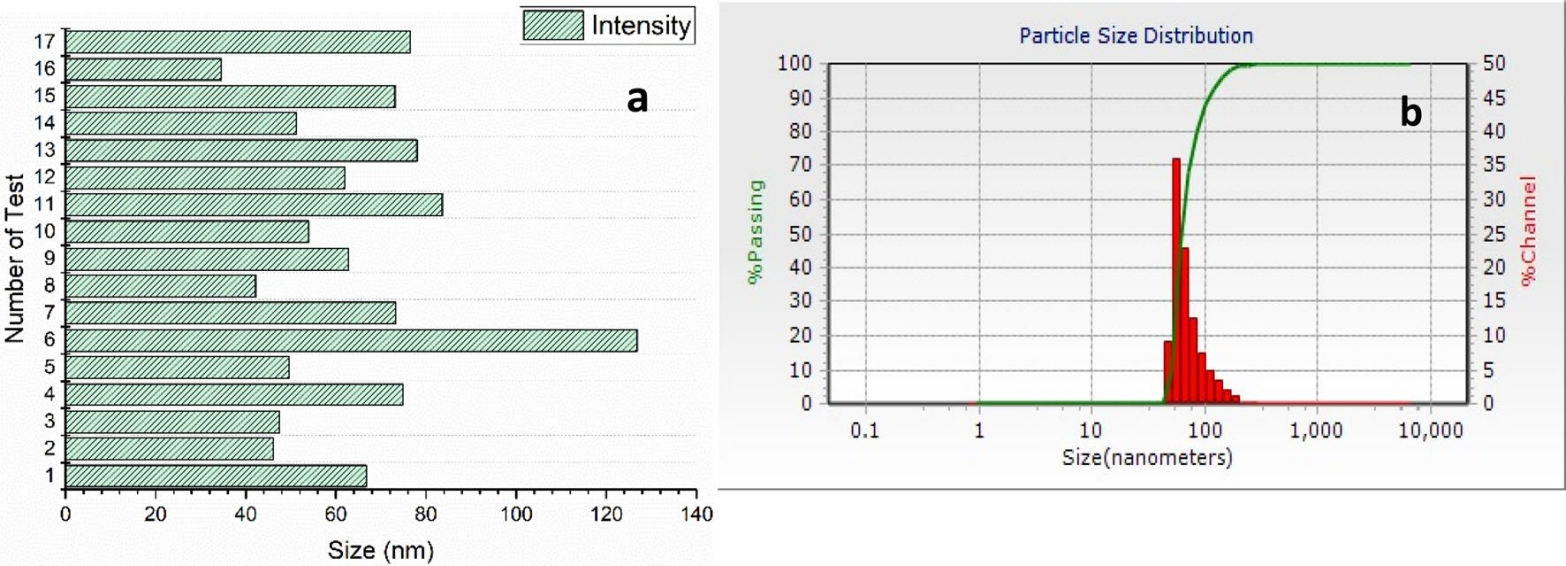
Comparative DLS of 17 synthesized samples (**a**) and DLS of sample **11** (**b**).

The particle size of nano-encapsulated phytosterols has been compared in (Fig. 4a). As mentioned earlier (UV–Vis), the highest absorbance of phytosterols is observed in capsules prepared according to run **11**. Although other runs have shown smaller sizes of capsules, particle size in Run **11** has remained below 100 nm and the polydispersity index of the obtained nanocapsules from Run **11** is in the suitable range (PDI˂ 0.7). So, it could meet our goal of producing capsules on a nanometer scale with the highest loading. The DLS of sample **11** has been shown in (Fig. 4b). The nanocapsules presented a mean diameter of 63 ± 38 nm and their particle size distribution is narrow which gives them more dispersibility, uniformity, and solubility. Positive zeta potentials (43.8 ± 0.25 mV) indicate an adequate homogeneity of the colloid and confirm its electrostatic stability and prevent the increase in size.

**Figure 5:**
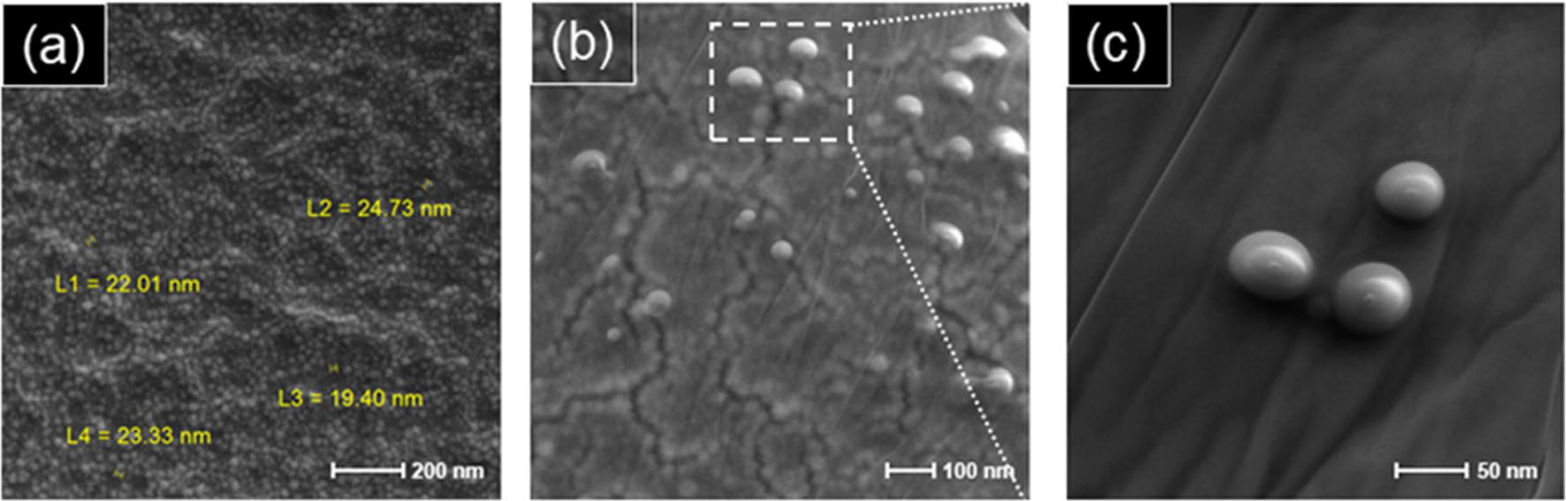
SEM images of the optimum SLN product (**a**–**c**).

According to the SEM images (Fig. 5), the product morphology in optimum conditions formed as spherical nanoparticles. As can be seen, the size of synthesized SLNs in sample **11** is below 100 nm. The polydispersity index (PDI) as an indicator of colloid stability is used to determine the standard deviation of the particle size distribution with the average hydrodynamic particle diameter. The PDI value of the obtained nanocapsules from run **11** is 0.4 as seen in Fig. 5a.

**Figure 6:**
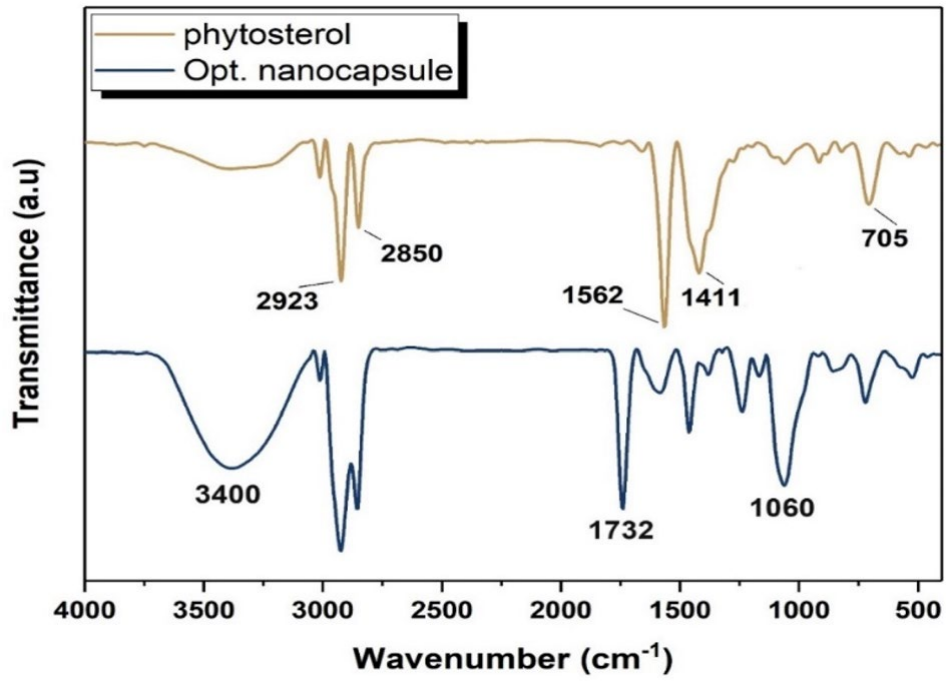
FT-IR spectra of extracted phytosterols and optimized nanocapsules.

FT-IR spectra of the extracted phytosterols and optimized nanocapsules are represented in (Fig. 6).

Extracted phytosterols exhibited characteristic bands as the following: 2923 and 2850 cm^-1^ for asymmetric stretching vibrations of C–H bonds in aliphatic groups, 1562 cm^-1^ for C=CH–CH_2_, 1411 cm^-1^ for –CH_2_ bending vibrations, and from 1000 to 1250 cm^-1^ for secondary C–O vibration in the C–O–H group^33^. The characteristic peaks of the phytosterols were found in the nanocapsules. The interaction between the phytosterols during the nanoencapsulation process could change the intensity of the characteristic peaks. The strong, broad absorption peak at about 3400 cm^−1^ could be related to O–H stretching. However, pure phytosterols showed a lower peak intensity in this region compared to the nanocapsules containing more hydroxyl groups. Further, the nano-encapsulated phytosterols had additional absorption peaks at 1732 and 1060 cm^−1^. These peaks could be attributed to the stretching vibration of C=O and C–O–C in the fat part and lecithin of SLNs^34,35^. Therefore, the nanoencapsulation of phytosterols has been shown by functional groups in the product.

**Figure 7:**
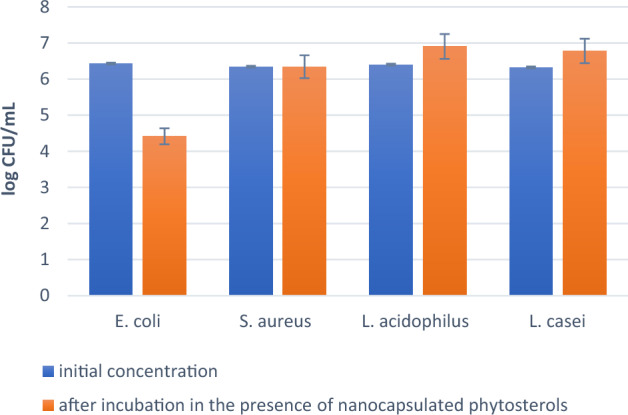
Antibacterial activity of nano-encapsulated phytosterols against different bacteria strains. This test was performed with a significance level of P < 0.01 with ANOVA analysis.

**Figure 8:**
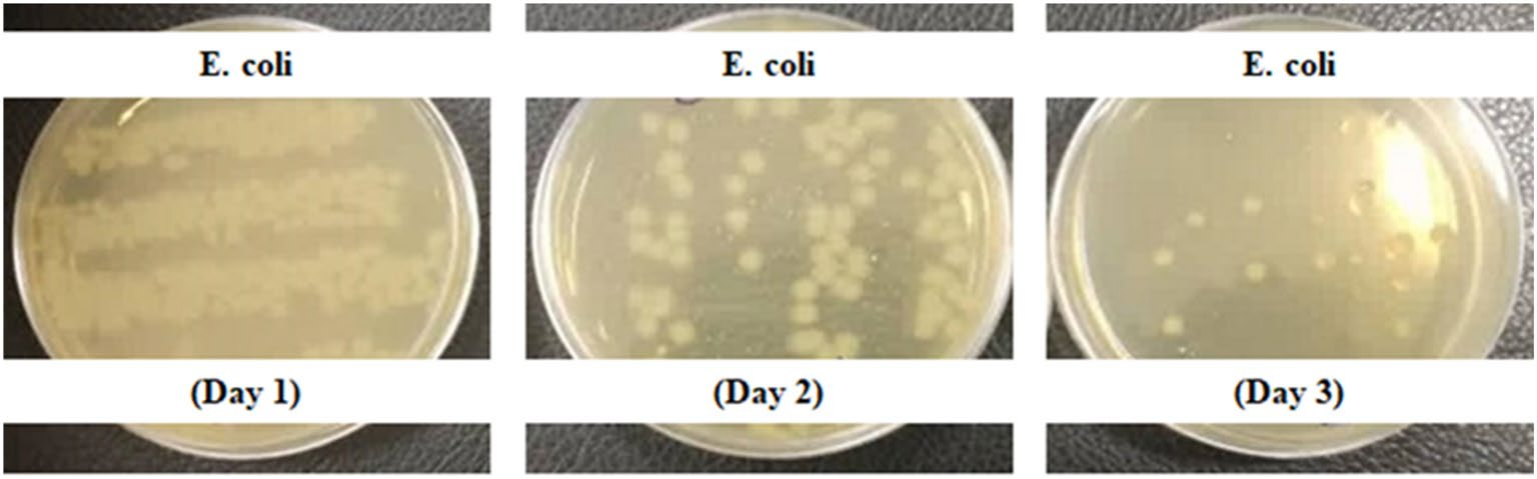
Inhibition and a reducing effect of nano-encapsulated phytosterols against *E. coli.*

The graphical illustration of antibacterial activity (log_10_ CFU/mL) of nano-encapsulated phytosterols against *E. coli, S. aureus*, *L. acidophilus,* and *L. casei* has been shown in (Fig. 7). It indicated the inhibited amount of CFU value of bacteria in the presence of nano-encapsulated phytosterols compared to the initial concentrations. It can be observed the nano-encapsulated phytosterols at the concentration of 100 µg/mL could inhibit the growth of *E. coli* (gram-negative) following 72 h incubation while in the case of *S. aureus* no significant inhibition effect on bacterial growth. However, nano-encapsulated phytosterols have been able to improve the growth of *L. acidophilus* and *L. casei* (as two conventional probiotics). As various factors affect the survival of probiotics, it seems that phytosterols have an important role in regulating the fluidity and permeability of cell membranes through the metabolic process and increasing the survival of *L. casei* and *L. Acidophilus*^36^. Some studies reported that high amounts of phytosterols have reduced the survival of probiotics^37,38^. Therefore, the controlled amount of phytosterols in encapsulated nanoparticles is very important to increase the survival of probiotics or antibacterial activity against pathogens^39^. In addition, the survival of probiotics in a lipid medium (the phase of nano-encapsulated phytosterols) is higher than in an aqueous medium due to the limited penetration of acid (H^+^ ions) and oxygen to their cell wall membrane^40^. Also, it seems that phytosterols’ antibacterial effect on gram-negative bacteria (*E. coli*), is related to their cell wall structure (lipopolysaccharides), while the gram-positive bacterial membrane (peptidoglycan) has a high polarity, so non-polar SLN containing phytosterols cannot be effective on them (*S. aureus*). The antibacterial activity of nano-encapsulated phytosterols against *E. coli* strains is shown in (Fig. 8) at different time intervals within 3 days.

**Figure 9:**
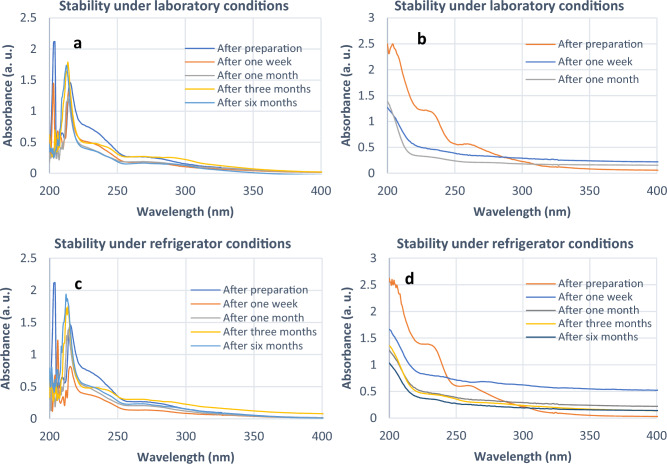
Stability comparison of nano-encapsulated phytosterols (**a** and **c** under laboratory and refrigerator respectively) versus pure phytosterols (**b** and **d** under laboratory and refrigerator respectively).

The potential alteration of phytosterols' chemical composition, particularly in ordinary circumstances (via oxidation), prompted our interest in examining the endurance of two variations of phytosterols (pure and covered) in relation to their UV absorbance. The stability of the extracted phytosterols compared with the nano-encapsulated phytosterols was investigated using UV–Vis analysis at 208 nm (Fig. 9) which is the maximum absorption wavelength of extracted phytosterols^41^. It was found that the stability of nano-encapsulated phytosterols was higher than pure phytosterols under all studied conditions (laboratory condition with normal humidity at room temperature, and 4 ℃ refrigerator condition with normal humidity over a period of six months) which may be due to the similar polarities of SLN and phytosterols with long-chain alkyl groups to produce stronger Vander Waals interactions between them or anti-oxidant property of ostrich oil^42,43^. Consequently, SLNs are a good coating to protect the phytosterols from environmental factors.

**Figure 10:**
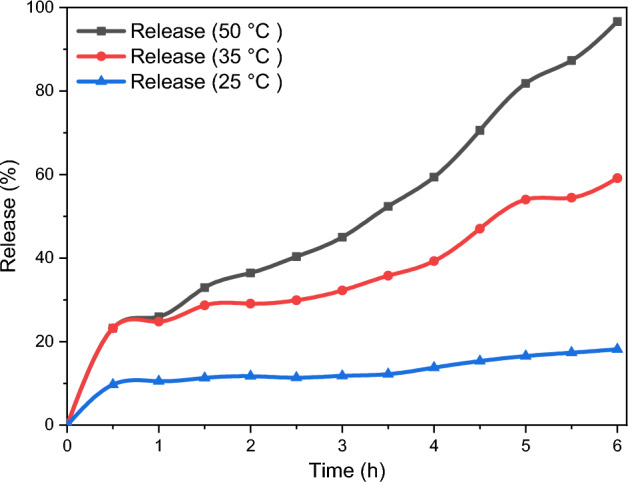
Cumulative release of extracted phytosterols.

Considering the lipidic composition of SLNs, they display a remarkable sensitivity to temperature variations, thereby requiring precise temperature monitoring during their release process. To investigate the impact of temperature on the liberation of the pharmaceutical compound, the nano-encapsulated phytosterols were subjected to examination at three distinct temperatures. The delivery of plant sterols at a temperature of 25 °C, as illustrated in Fig. 10, demonstrated a nearly uniform decrease, falling below 20%. As a result, it is possible to maintain the phytosterols at room temperature while preserving a significant portion. Furthermore, the release of plant sterols was observed to occur with a significant increase when exposed to a temperature of 50 °C. Consequently, the nanocapsules exhibit a regulated release at ambient temperature in contrast to the higher temperatures, which can plausibly be attributed to the alterations in the structural composition of the SLN membrane. Hence, stable SLNs possess the capability to be stored for a duration of six months at ambient temperature without any occurrence of coagulation.

## Conclusion

Plant sterols were extracted from flaxseed oil by caffeine under mild and simple conditions to avoid any changes in the structure of phytosterols which is common in the saponification method because of high temperature. Ostrich oil and sheep fat nanocapsules (SLN) containing phytosterols were successfully produced by a natural surfactant (Soy lecithin) through the co-sonicated coacervation technique. This novel method can prevent intrinsic coagulation during the formation of SLNs and control the size of particles as well as increase loading. The optimum product morphology was spherical and uniform, with an average size lower than 100 nm. According to BBD, all the found trends showed well correlation with the phytosterols content of SLN and particle size of nanocapsules. Optimal values showed that the ratio of ostrich oil to sheep fat should be at least double which is much more beneficial for the body. According to the calculations, the amount of phytosterol has the highest impact factor and 0.06 g of it is sufficient to form nanocapsules. However, glucose as a factor that can reduce nanoparticle aggregation should be approximately close to the maximum value. Increasing the stirring time and the surfactant (lecithin) don’t have much effect on the results. So, the minimum amount of lecithin (6%) and 2 h are suitable to make a homogeneous mixture. The encapsulation efficiency (85.46%) was good under optimum conditions. Also, the extremely positive zeta potential value (43.8 ± 0.25 mV) leads to larger repulsive forces and prevents aggregation of the particles. The excellent antibacterial activity of nano-encapsulated phytosterols against *E. coli* has shown there can be an effective interaction between SLN and negative gram bacteria. It was found that the stability of nano-encapsulated phytosterols was higher than extracted phytosterols under all studied conditions due to the encouraging stability and versatility of lipid-based nanocarriers. Also, the nano-encapsulated phytosterols have shown controlled release at ambient temperature.

Overall, nanoencapsulation of lipophilic ingredients, can be used in a variety of industries including pharmaceuticals, cosmetics, and food. It results in enhanced resistance of them to light, oxygen, and moisture, thereby improving their stability. Additionally, nanoencapsulation leads to increased solubility of lipophilic materials, preventing any deterioration or precipitation that may compromise their quality. The innovation of this work is a novel extraction and the use of edible and natural components to make a stable colloid with the goal of green chemistry which is designing new products by reducing the use of hazardous substances to nanoencapsulation. For the first time, the coacervation method was reinforced by ultrasound waves to produce nanoparticles with a narrow size distribution. In this way, we could resolve the problems of SLNs by co-sonicated coacervation and Box–Behnken optimization which is essential to show the impact of the chemical variables on the drug entrapment efficiency and particle size. In the forthcoming works, it is feasible to utilize lipophilic substances, such as food flavorings, aromas, terpenes, or antioxidants, as an alternative to phytosterols, in addition to other effective components.

## Materials and methods

### Materials and apparatus

The FT-IR spectra were performed by a KBr disk technique at room temperature (FT-IR 410, Jasco Inc Easton, Maryland, USA) ranging from 4000 to 400 cm^-1^. In this method, to prepare a sample pellet with a diameter of 13 mm, a small percentage of the sample (ranging from 0.1 to 1%) is mixed with 200 to 250 mg of powdered KBr. Afterward, the selected pellet is crushed into a fine powder and then placed into a die to shape it into a pellet form. Moreover, a significant amount of pressure, reaching up to 8 tons, is applied to the sample in a vacuum for several minutes to create a transparent pellet. Additionally, any residual air and water molecules attached to the pellet are removed through a process called degassing^44^. A Field Emission Scanning Electron Microscope (FESEM, SIGMA VP-500, ZEISS company Germany) investigated the surface morphologies of the nanocapsules. The phytosterols concentration was assessed from 200 to 400 nm using a UV–Vis spectrophotometer (Perkin Elmer/Lambada 25 UV/Vis spectrophotometer, USA). The particle size distribution histograms of nano-encapsulated phytosterols were carried out by Dynamic Light Scattering (DLS) by a Zeta sizer nano instrument (Malvern Instruments, Nano Zs, ZEN 3600, UK) working with a laser 532. The reaction mixture was sonicated in a QS 1C ultrasonic bath. A Shimadzu GC-9A connected to a hydrogen flame ionization detector (FID) analyzed the composition of the achieved phytosterols. A DB-1 glass capillary column (30 × 0.25 mm) was used for separation. The column operating conditions were as follows: helium was a carrier gas at a flow rate of 1 mL/min with a split ratio of 1:50; the temperature of 305 °C was considered for the detector and the gasifying chamber; the inlet temperature was 280 °C, and the column temperature was increased from 150°C to 320°C at 5°C/min. Component identification of nanocapsules was done on a BBD and accomplished using Minitab Version 16. A Milli-Q system (Millipore, Bedford, MO) was used to obtain deionized water. The antibacterial effect (in vitro) was assessed against *Escherichia Coli* (ATCC 10,538) and *Staphylococcus aureus* (ATCC 6538) as food-borne bacteria and the effect of nano capsulated phytosterol was evaluated on *Lactobacillus acidophilus* (ATCC 15,708), *Lactobacillus casei* (ATCC 393) as probiotic bacteria: through colony forming units (CFU) count method. These bacteria were purchased from the Pasteur Institute of Iran.

Flaxseed oil, caffeine, ethanol, and lactic acid were prepared by Sigma company without any further purification. Soy lecithin, sheep fat, ostrich oil, and glucose were purchased from Kimia Exir (an Iranian company).

### Extraction method

About 5 g of flaxseed oil in 20 mL ethanol, were added drop-wise to a supersaturated solution of caffeine (67 mg/mL H_2_O). The resulting mixture was stirred (600 rpm) for a sufficient time at 60°C to completely dissolved, and allowed to cool at room temperature. It was filtered and the solution was heated using a rotary evaporator (with a mild speed and below 50 °C) until precipitation occurred. This process was repeated (three times) and the resulting raw non-saponifiable components were washed twice with warm H_2_O (below 50 °C) and dried in a desiccator. The quantity of phytosterols obtained from this method corresponds to the highest measurement (400 mg/100 g oil) reported in previous publications^26^.

### Nanoencapsulation of phytosterols

Nanocapsules were prepared by the coacervation technique and carried out using two natural lipids (sheep fat and ostrich oil) in different proportions (mentioned in (Table 1)) to find the optimal SLN formulation. The micellar solution of the fatty acids alkaline salt (soap) was prepared by adding a stoichiometric amount of NaOH ethanolic solution to the ethanolic solution of two lipids mixture (200 mg). After that, it was dispersed in a lecithin aqueous solution (with different concentrations in (Table 1)) and the mixture was heated in an ultrasonic bath above the Krafft point (50 °C) to obtain a clear soap micellar solution. 0.06 g phytosterols in 2 mL of EtOH and glucose solutions was then added and it was placed under magnetic stirring for 2h to obtain a homogeneous mixture. A lactic acid solution (coacervating solution) was added drop-wise in an ultrasonic bath until the pH reached below the isoelectric point (4.0). Then, the obtained colloid was rapidly cooled to 15 °C in a water bath for better formation of nanocapsules under stirring at 300 rpm to stabilize colloid. The SLNs were filtered and dried with a freeze-dryer (72 h at – 70 °C).

### Evaluation of anti-microbial activity

The antibacterial activity of extracted phytosterols was calculated on Bovine Serum Albumin (BSA) using the colony forming units (CFU) count method^27^. The bacterial strains of Escherichia coli, Staphylococcus aureus, Lactobacillus acidophilus, and Lactobacillus casei were grown on nutrient agar at 37 °C for 18 to 24 h and suspended in a saline solution to yield a bacterial suspension of 2.7 × 10^6^ CFU/mL for Escherichia coli, 2.2 × 10^6^ CFU/mL S. aureus, 2.5 × 10^6^ CFU/mL for Lactobacillus acidophilus and 2.1 × 10^6^ CFU/mL for Lactobacillus casei. The cell density was also settled on 0.5 MacFarland turbidity standard. The standardized inoculum was shifted and spread on BSA plates using sterile swabs. For 3–5 days, the plates underwent incubation at 37 °C. For each strain, three repetition steps were performed. The plates were determined for bacterial growth inhibition, indicating the number of CFU/mL. This takes into account all of the dilutions of the samples.

### Drug release behavior

The extracted nano-encapsulated phytosterols were immersed in 50 mL phosphate buffer saline at pH = 7.4, and using Beer-Lambert's law, the calibration curve was verified in PB (phosphate buffer). In beakers of 30 mL phosphate buffer saline, the tubes were then immersed, sealed, and located in an incubator at 25 °C, 37 °C, and 50 °C. During six hours for every half-hour interval, the release profiles were investigated.

### Stability of the nano-encapsulated phytosterols

The stability of the extracted phytosterols compared with the nano-encapsulated phytosterols under laboratory conditions with normal humidity at room temperature, and 4 ℃ refrigerator conditions with normal humidity at 4 °C over a period of six months was investigated using UV–Vis analysis.

### Experimental design methodology

In the current research, BBD was applied to optimize and assess the impact of independent variables (sheep fat to ostrich oil proportion (X_1_: 1.0–2.0), percentage of glucose (X_2_: 40–60), amount of phytosterols (X_3_: 0.06–1.0), percentage of lecithin (X_4_: 6.0–10.0) and nanoencapsulation time (X5: 2.0–5.0) on the efficiency and particle size. Box–Behnken is rotatable, spherical, or nearly rotatable including a central point with the midpoints of the variable space edges. The number of experiments (N) needed for the development of BBD can be considered as Eq. (3):

3 $$ N = 2k\left( {k - 1} \right) + C_{0} $$

C_0_ is the number of central points and k is the number of factors. So, 17 runs were performed to optimize the five variables at three low, medium, and high levels in the current BBD. The design center point was replicated in triplicate to estimate the error. The (Table 2) shows the Box–Behnken matrix and the responses. The experiments were done randomly to minimize the impacts of unexplained variability in the detected responses. The generalized type of the second-order polynomial equation is given below: (Eq. (4))

4 $$ Y = \beta_{0} + \sum {\beta_{i} } X_{i} + \sum {\beta_{ii} } X_{i}^{2} + \sum {\beta_{ij} X_{i} X_{j} + \varepsilon } \;\;\;\;\;\;\;i = {1}:{5},j = {1}:{5} $$

*Y* and* X*_*i*_ are respectively the anticipated response and the independent variable. The *X*_*i*_^*2*^ represents the quadratic and *X*_*i*_*X*_*j*_ is used for interaction terms. *β*_*i*_*, β*_*ii*_ and *β*_*ij (i≠j)*_ are respectively the coefficients of linear, quadratic, and interaction. While *β*_*0*_ and *ε* respectively indicate the constant and the random error.

### Supplementary Information

Supplementary Figures.

## Data Availability

The datasets used and/or analyzed during the current study are available from the corresponding author upon reasonable request.
