# Supplementary material for: Co-sonicated coacervation for high-efficiency green nanoencapsulation of phytosterols by colloidal non-biotoxic solid lipid nanoparticles
Source: Sci Rep. 2024 Feb 26;14:4671. doi: 10.1038/s41598-024-54178-7 (PMC10897223; doi:10.1038/s41598-024-54178-7)
Supplement: Supplementary file 1 — Supplementary Figures. [file 41598_2024_54178_MOESM1_ESM.docx]

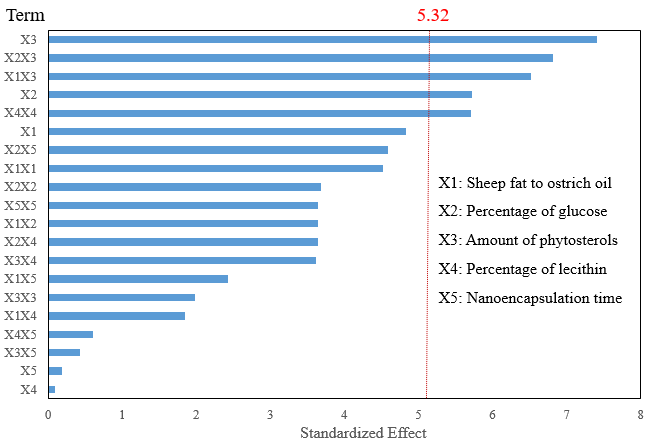


**Supplementary Figure 1: Pareto charts of the standardized effects**





**Supplementary Figure 2: Calibration of the phytosterols solution**
